# Endothelial-specific depletion of TGF-β signaling affects lymphatic function

**DOI:** 10.1186/s41232-021-00185-4

**Published:** 2021-12-01

**Authors:** Kunpei Fukasawa, Kako Hanada, Kei Ichikawa, Masanori Hirashima, Takahiro Takagi, Susumu Itoh, Testuro Watabe, Fumiko Itoh

**Affiliations:** 1 Laboratory of Cardiovascular Medicine, Tokyo University of Pharmacy and Life Sciences, 1432-1, Horinouchi, Hachioji, Tokyo, 1925-0392 Japan; 2 Laboratory of Stem Cell Regulation, Tokyo University of Pharmacy and Life Sciences, 1432-1, Horinouchi, Hachioji, Tokyo, 1925-0392 Japan; 3 Division of Pharmacology, Niigata University Graduate School of Medical and Dental Sciences, 1-757 Asahimachi-dori, Chuo-ku, Niigata, 951-8510 Japan; 4 Laboratory of Biochemistry, Showa Pharmaceutical University, 3-3165, Higashi-Tamagawagakuen, Machida, Tokyo, 194-8543 Japan; 5 Department of Biochemistry, Graduate School of Medical and Dental Sciences, Tokyo Medical and Dental University, 1-5-45 Yushima, Bunkyo-ku, Tokyo, 113-8549 Japan

**Keywords:** TGF-β, Lymphatic vessel, Endothelial cell, Prox1, Tumor metastasis

## Abstract

**Background:**

Transforming growth factor (TGF)-β is a multifunctional cytokine involved in cell differentiation, cell proliferation, and tissue homeostasis. Although TGF-β signaling is essential for maintaining blood vessel functions, little is known about the role of TGF-β in lymphatic homeostasis.

**Methods:**

To delineate the role of TGF-β signaling in lymphatic vessels, TβRII^fl/fl^ mice were crossed with Prox1-Cre^ERT2^ mice to generate TβRII^fl/fl^; Prox1-Cre^ERT2^ mice. The *TβRII* gene in the lymphatic endothelial cells (LECs) of the conditional knockout TβRII^iΔLEC^ mice was selectively deleted using tamoxifen. The effects of *TβRII* gene deletion on embryonic lymphangiogenesis, postnatal lymphatic structure and drainage function, tumor lymphangiogenesis, and lymphatic tumor metastasis were investigated.

**Results:**

Deficiency of LEC-specific TGF-β signaling in embryos, where lymphangiogenesis is active, caused dorsal edema with dilated lymphatic vessels at E13.5. Postnatal mice in which lymphatic vessels had already been formed displayed dilation and increased bifurcator of lymphatic vessels after tamoxifen administration. Similar dilation was also observed in tumor lymphatic vessels. The drainage of FITC-dextran, which was subcutaneously injected into the soles of the feet of the mice, was reduced in TβRII^iΔLEC^ mice. Furthermore, Lewis lung carcinoma cells constitutively expressing GFP (LLC-GFP) transplanted into the footpads of the mice showed reduced patellar lymph node metastasis.

**Conclusion:**

These data suggest that TGF-β signaling in LECs maintains the structure of lymphatic vessels and lymphatic homeostasis, in addition to promoting tumor lymphatic metastasis. Therefore, suppression of TGF-β signaling in LECs might be effective in inhibiting cancer metastasis.

**Supplementary Information:**

The online version contains supplementary material available at 10.1186/s41232-021-00185-4.

## Background

Transforming growth factor-β (TGF-β) is a secreted dimeric protein that has pleiotropic effects and plays a key role in many cellular processes during both embryogenesis and tissue homeostasis in adults [1]. Therefore, abnormal TGF-β signaling has also been associated with various diseases, including cancer, fibrotic disorders, and cardiovascular diseases [2] [3]. The TGF-β signaling pathway is initiated through two different serine/threonine kinase receptors: type II (TβRII) and type I (TβRI; also termed activin receptor-like kinase-5 [ALK5]). In canonical TGF-β signaling, the activated receptor complex phosphorylates receptor-regulated Smads (R-Smads; i.e., Smad2 and Smad3) at two serine residues at their C-terminal to permit the phosphorylation of two R-Smads to form ternary complexes with the common partner Smad (Co-Smad), Smad4. The R-Smad/Co-Smad complex then enters the nucleus where it acts as a transcriptional factor to regulate the expression of TGF-β target genes in cell type-specific and context-dependent manners [4].

Lymphatic vessels maintain homeostasis by balancing tissue fluid throughout the body, regulating inflammation via the immune system, forming new lymphatic vessels from preexisting lymphatic vessels upon environmental stimulus, and contributing to tumor metastasis [5]. Lymphatic progenitor cells originate from the venous endothelial cells during embryonic development. The expression of the transcriptional factor Prox-1 is imperative for the development and maintenance of lymphatic endothelial cells (LECs). Prox1, a master regulator of LECs, induces the expression of vascular endothelial growth factor receptor 3 (VEGFR3) in the lymphatic progenitor cells that bud from the cardinal vein. Subsequently, the lymphatic progenitor cells form the lymph sac in a VEGF-C/VEGFR3 dependent manner. The VEGF-C/VEGFR3 pathway plays a central role in spreading the lymphatic network throughout the body [6, 7]. Although the molecular mechanisms that control lymphangiogenesis have been elucidated, little is known about the mechanisms that maintain the plasticity and stability of lymphatic vessels.

Mouse genetic studies regarding the deficiency of TGF-β signaling in fetal LECs have revealed a significant reduction in lymphatic vessel germination and remodeling in the absence of TGF-β [8]. Contrastingly, TGF-β inhibited LEC differentiation through suppression of Prox1 and LYVE-1 expression in cultured LECs. Consistently, the blockage of endogenous TGF-β signaling by a chemical inhibitor enhanced lymphangiogenesis in a mouse model of chronic peritonitis [9]. Thus, the nature of involvement of TGF-β signaling in lymphangiogenesis is still unclear.

In this study, we showed that TGF-β signaling plays a key role in maintaining the structure and function of lymphatic vessels. LEC-specific deletion of the *TβRII* gene results in dilation of the lymphatic lumen and impaired lymphatic drainage function. When Lewis lung carcinoma cells constitutively expressing green fluorescent protein (LLC-GFP) were implanted into mice lacking the *TβRII* gene in their LECs, tumor lymphatic vessels were dilated and lymphatic metastasis was suppressed. These results indicate that TGF-β signaling acts as a tumor malignant factor via lymphatic endothelial cells.

## Materials and methods

### Mice

TβRII^fl/fl^ mice [10] were crossed with Prox1-Cre^ERT2^ mice [11] to generate TβRII^fl/fl^; Prox1-Cre^ERT2^ mice. Tamoxifen (Tx) (Sigma-Aldrich, St. Louis, MO T5648) dissolved in corn oil (20 mg/mL) was intraperitoneally administrated to mice (40 mg/kg/day) for consecutive 5 days into control (TβRII^fl/fl^) mice and TβRII^fl/fl^; Prox1-Cre^ERT2^ mice, where TβRII gene in LECs is deficient (TβRII ^iΔLEC^). For embryonic analysis, pregnant mice were administrated with Tx (40 mg/kg/day) 10.5 and 11.5 days after mating. Then, mice were sacrificed to analyze embryos at E13.5. The lymphatic structures of adult mouse ear and tail were examined 4 weeks after the first injection of Tx. The mice were housed in the animal facilities of the Laboratory Animal Resource Center at the Tokyo University of Pharmacy and Life Sciences under specific pathogen-free (SPF) conditions at constant temperature and humidity and fed a standard diet. Treatment of the mice was in accordance with the institutional guidelines of the Animal Care and Use Program of the Tokyo University of Pharmacy and Life Sciences (L18-3, L19-18, L20-5).

### Establishment LLC cells expressing eGFP

LLC cells were cultured in Dulbecco’s modified Eagle’s medium (DMEM; Nakalai Tesuque, Kyoto, Japan) containing 10% fetal calf serum (FCS; Invitrogen, Carlsbad, CA, USA), 1× MEM nonessential amino acids (NEAA; Sigma-Aldrich) and 100 U/mL penicillin/streptomycin (FUJIFILM Wako, Osaka, Japan) [12]. To obtain the eGFP stable transformants, LLC cells were co-transfected with pEGFP-N1 (GenBank Accession #U55762) and CS-CDF-CG-PRE (RDB04379) including the Zeocin-resistant gene. For selection of stable transformants, LLC cells were maintained in DMEM containing 600 mg/mL of Zeocin (Invitrogen).

### In vivo lymphatic drainage of FITC-dextran

Four weeks after Tx administration, male TβRII^fl/fl^ and TβRII ^iΔLEC^ mice (7-12 weeks old) were subcutaneously injected with 50 μl of 10 mg/mL FITC-dextran (MW 2,000,000, Sigma-Aldrich, FD2000S) into their rear footpads. Five minutes later, the transport of FITC-dextran was visualized with MVX10 fluorescence stereomicroscope (Olympus, Tokyo, Japan).

### In vivo xenografts

Male TβRII^fl/fl^ and TβRII ^iΔLEC^ mice (7–12 weeks old) were subcutaneously implanted with LLCs (2.5 × 10^5^ cells) in 100 μl of PBS. Tumor volumes (V) were calculated using the following formula: length × width × width × 0.5 [13]. The tumors were surgically removed and embedded into a frozen section compound (Leica). Lymphatic metastasis was measured by the expression of eGFP when LLC-GFP cells implanted into mouse footpad were metastasized. In brief, the legs were clarified with CUBIC 1 week after the LLC-GFP transplantation [14] and tumor metastasis to the popliteal lymph nodes (PLN) was evaluated with the fluorescent stereomicroscope (Olympus).

### Immunofluorescence analysis and quantification

Antibodies were obtained from the following sources: Rabbit polyclonal anti-LYVE-1 (ab14917) and anti-Ki-67 (ab15580) antibodies from Abcam (Cambridge, UK); rat monoclonal anti-PECAM-1 (550274) and anti-VE-cadherin (550548) antibodies from BD Transduction Laboratories (Franklin Lakes, NJ); goat anti-VEGFR3 antibody (35917) from R&D systems (Minneapolis, MN). Ear skins from adult mice were dissected, fixed in 4% paraformaldehyde (PFA) in PBS for overnight at 4 °C, and washed three times with PBS. After connective tissues and hairs were removed, the samples were permeabilized in 0.1% Triton X-100 (Sigma-Aldrich) and incubated in blocking reagent (Dako, Glostrup, Denmark) before staining. The tumors and 1 cm from buttock of the tail were surgically removed and embedded into a frozen section compound (Leica). Fresh frozen sections (5 μm) were cut with a CM1850 cryostat (Leica Camera AG, Wetzlar, Germany), mounted on Cryofilm (Leica), and fixed in 100% ethanol and 4% PFA. The films were washed three times with PBS, permeabilized with 0.1% Triton X-100 for 5 min, and blocked with Blocking Reagent for 1 h at 37 °C. First antibodies in Blocking Reagent were added and incubated overnight at 4 °C. The films were washed three times with PBS and then incubated with Alexa488-conjugated donkey anti-rabbit IgG (A21206, Thermo Fisher Scientific, Waltham, MA), Alexa488-conjugated goat anti-rabbit IgG (A11008, Thermo Fisher Scientific), Alexa594-conjugated goat anti-rat IgG (A21208, Thermo Fisher Scientific) antibody, Alexa594-conjugated donkey anti-rat IgG (A21209, Thermo Fisher Scientific) antibody, or Alexa647-conjugated donkey anti-goat IgG (A11058, Thermo Fisher Scientific) at 1:200 for 1 h at room temperature [13]. After the nuclei as needed were stained with 2 μg/mL DAPI (Dojindo Laboratories, Kumamoto, Japan) for 5 min, the samples were washed three times with PBS, and the fluorescence signals were visualized by BZ-9000 fluorescence microscope (Keyence, Osaka, Japan). For mouse ear skin, the LYVE-1-positive area and the number of branches were analyzed with 12 μm^2^ image from each of the 4 mice used. For tumor sections, the immunostaining-positive area was measured using three independent images at 10x magnification from each mouse (3 control and 6 TβRII^iΔLEC^ mice). For tail sections, the immunostaining-positive area was measured using one image including tissues around the artery at × 10 magnification from each of the 4 mice. These analyses were carried out using BZ-X analyzer imaging software (Keyence) or ImageJ. The analysis of proliferative lymphatic endothelial cells from each mouse (*n* = 3) were measured the rate of Ki-67-positive cells from at least 100 VEGFR3-positive LECs per mouse.

### RNA isolation and RT-PCR

Total RNA was extracted using a NucleoSpin® RNA Plus kit (Takara Bio Inc., Shiga, Japan). Reverse transcription was performed with a PrimeScript II 1st strand cDNA Synthesis Kit (Takara). qPCR was performed with a KAPA SYBR Fast qPCR kit (Nippon Genetics, Tokyo, Japan). All reactions were carried out on a LightCycler®96 (Roche). Each sample was analyzed in triplicate at least twice for each PCR measurement. Melting curves were checked to ensure specificity. Relative quantification of mRNA expression was calculated using the standard curve method with the GAPDH level. Before qPCR, the DNA fragment amplified using each primer set was detected to be a single band with the correct size by agarose gel electrophoresis. The following primer sets were used to amplify cDNAs; 5′-CAGCCCACCCTCAATACCAG-3′ and 5′-AGAAGGTGTTTGTGGCTGCT-3′ for mouse VEGF-C [15], 5′-TGCAGTGGCAAAGTGGAGATT-3′ and 5′-TGCCGTTGAATTTGCCGT-3′ for mouse GAPDH [16].

### Statistical analysis

Numerical results were expressed as means ± standard deviation. Significance was assessed using the unequal variances *t* test and the chi-square test. Probability values below 0.05 were considered significant.

## Results

### TGF-β regulates lymphatic network development

**Fig. 1:**
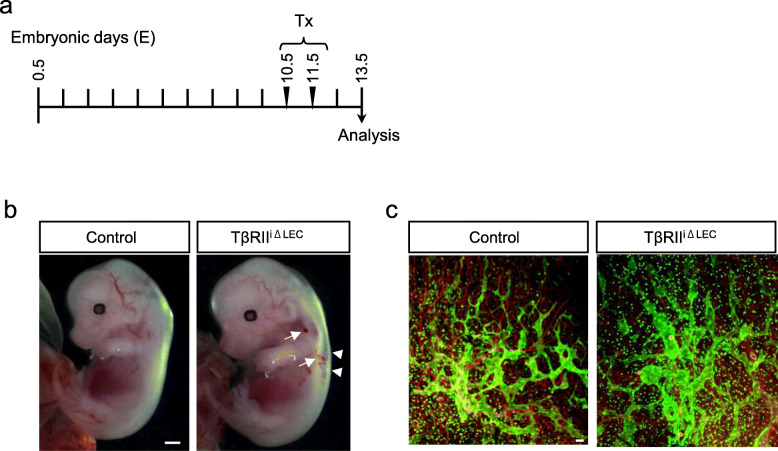
TβRII^iΔLEC^ mice exhibit embryonic edema and abnormal lymphatic vessel structure. **a** Treatment protocol for i.p. administration of tamoxifen (Tx). Pregnant mice were injected with Tx at E10.5 and E11.5, and embryos were analyzed at E13.5. **b** Gross analysis of TβRII^fl/fl^; Prox1-CreER^T2^ (TβRII^iΔLEC^), and littermate control (TβRII^fl/fl^) embryos at E13.5. Mild edema (arrowhead) and blood clots (arrows) are observed in TβRII^iΔLEC^ pups. Scale bar: 1 mm. **c**. Back skin immunohistochemistry of mice embryos at E13.5 with anti-LYVE-1 (green) and anti-PECAM-1 (red) antibodies. Scale bar: 100 μm

In mouse development, the initiation of lymphatic vessel differentiation is observed at embryonic day 10–10.5 (E10–10.5) in the anterior cardinal vein with a subpopulation of endothelial cells expressing LYVE-1, Sox18, Prox1, and VEGFR3 [6] [7, 17]. To verify the effect of TGF-β signal deficiency on active lymphangiogenesis of LECs, Tx was administered to pregnant mice 10.5 and 11.5 days after mating. Next, we analyzed the phenotype of embryos at E13.5 (Fig. 1a). Mild edema and blood clots were found on the back of TβRII^iΔLEC^ embryos harvested from Tx-treated pregnant mice (Fig. 1b). Therefore, we visualized lymphatic vessels in the skin from their backs using the anti-LYVE-1 antibody (Fig. 1c). Interestingly, lymphatic networks with abnormally dilated lymphatic vessels were observed in the TβRII^iΔLEC^ embryos. These results are consistent with previous findings in which TGF-β signaling acts as an active regulator of lymphangiogenesis [9].

### TβRII^iΔLEC^ mice injected with Tx showed expanded lymphatic vessel network

**Fig. 2:**
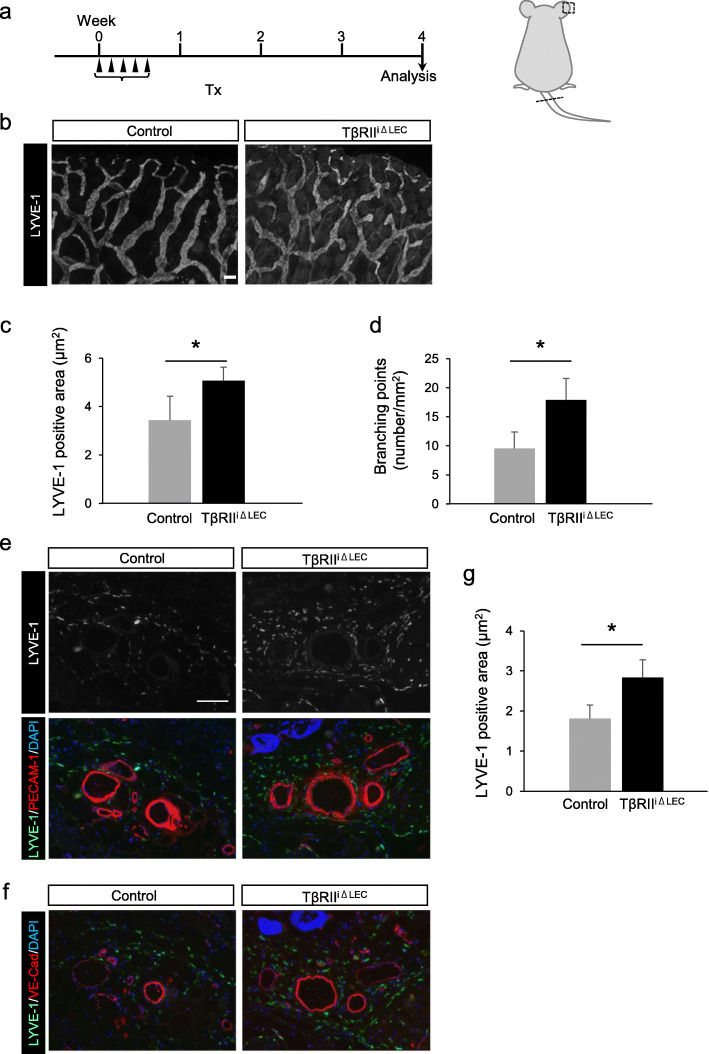
Lymphatic vessels increase in TβRII^iΔLEC^ mice. **a**. Experimental schedule. Mice were treated with i.p. tamoxifen (40 mg/kg) for five consecutive days and sacrificed at day 22 (3 weeks). The ears shown using the dotted square and the tail shown using the dotted line were analyzed. **b** Whole-mount LYVE-1 staining of ear skin from control and TβRII^iΔLEC^ mice. Representable images are shown. Scale bar: 100 μm. **c**. Image-based analysis of LYVE-1-positive lymphatic vessel area. The average of LYVE-1-positive area in TβRII^iΔLEC^ mice (*n* = 4) was significantly higher than that in control mice (*n* = 4). **d** Number of branching points of lymphatic vessels. The average of the branching number in TβRII^iΔLEC^ mice (*n* = 4) was significantly higher than that in control mice (*n* = 4). **e**, **f** Immunohistostaining of the transverse section of the mice tails with anti-LYVE-1 (green) and anti-PECAM-1 (red) (**e**)/VE-Cadherin (red) antibodies (**f**). Sections were counterstained with DAPI (nucleus, blue). Scale bar: 100 μm. **g** Image-based analysis of LYVE-1-positive lymphatic vessel area of Fig. 2e from control (*n* = 3) and TβRII^iΔLEC^ mice (*n* = 3)

The deficiency of TGF-β signaling in lymphatic vessels was studied using the ear skin and tail 4 weeks after postnatal mice were administered with Tx (Fig. 2a). To visualize the lymphatic vessels of the mouse ear [18], the lymphatic capillary maker, LYVE-1 was detected with fluorescence. Remarkable differences in the composition of the lymphatic vessel network were observed between control (TβRII^fl/fl^ mice treated with Tx) and TβRII^iΔLEC^ mice (Fig. 2b). In adult TβRII^iΔLEC^ mice, the lymphatic networks exhibited distorted structures with dilated and narrowed lymphatic vessels compared to the control mice. Their dilated structures were not as remarkable as those seen during embryogenesis. Furthermore, the LYVE-1-positive area (Fig. 2c) and the number of lymphatic branches in TβRII^iΔLEC^ mice (Fig. 2d) were significantly increased compared to those in the control mice.

The transverse sequential sections of mouse tails were stained with antibodies against LYVE-1 and PECAM-1, which is a marker of endothelial cells, or against LYVE-1 and VE-cadherin, which is an endothelial-specific adhesion molecule. There were no differences in the vessel structure stained with anti-PECAM-1 (Fig. 2e) or anti-VE-cadherin antibodies (Fig. 2g) of mouse tails between control and TβRII^iΔLEC^ mice, whereas the structures of the lymphatic vessels were increased in TβRII^iΔLEC^ mice compared with the control mice (Fig. 2e). When we measured the area of LYVE-1-positive lymphatic capillary, LYVE-1-positive LECs in TβRII^iΔLEC^ mice were significantly increased compared to those in the control mice (Fig. 2g). These results suggest that TGF-β signaling might control the number of LYVE-1-positive lymphatic endothelial cells.

### TβRII^iΔLEC^ mice treated with Tx showed expanded lymphatic vessel network

**Fig. 3:**
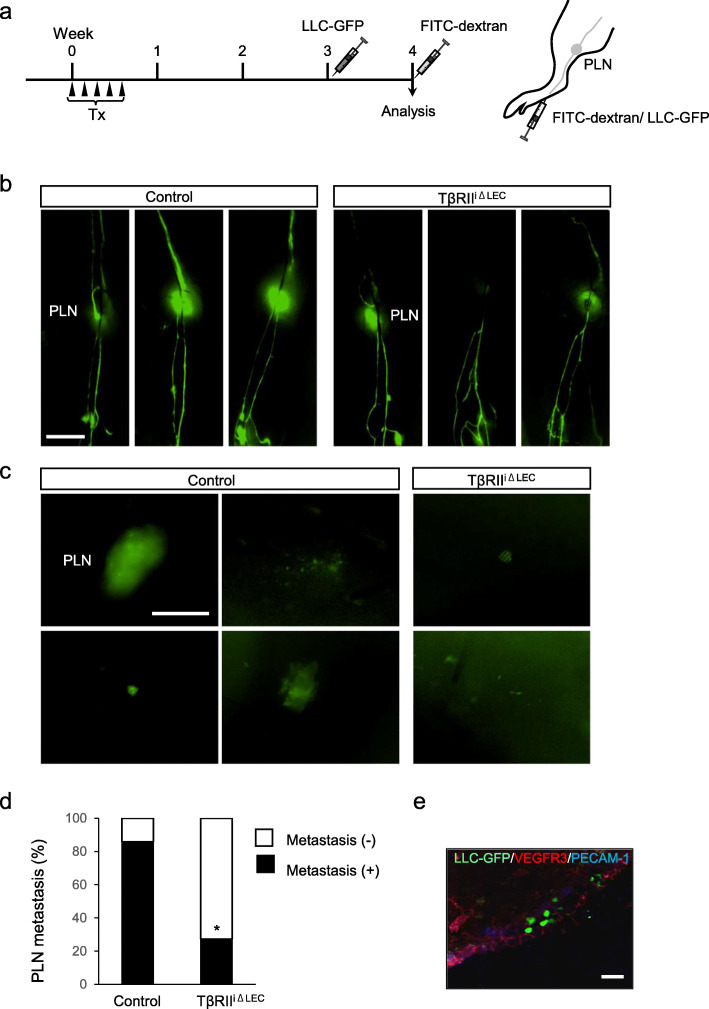
TβRII^iΔLEC^ mice exhibit decreased lymphatic drainage and tumor lymphatic metastasis. **a**. The mice were intraperitoneally administered Tx for five consecutive days. Three or 4 weeks later, LLC-GFP or FITC-dextran respectively were subcutaneously injected into their rear footpads independently. **b** Representative images of lymph flow from the lower extremities to the popliteal lymph node (PLN). (*n* = 7 mice per genotype) Scale bar: 5 mm. **c** Representative images of LLC-GFP metastasized to the PLN. Scale bar: 500 μm. **d** Quantification of metastasis (black) and no metastasis (white) to the PLN. Scale bar: 200 μm. (control; *n* = 7, TβRII^iΔLEC^; *n* = 11) **e** Immunohistostaining of the sagittal section of PLN from control mice with anti-VEGFR3 (red) and anti-PECAM-1 (blue) antibodies. Green fluorescence was derived from LLC-GFP. Scale bar: 100 μm

Next, we examined the lymphatic drainage function in TβRII^iΔLEC^ mice. It is known that any substrate injected into the hind footpad is excreted in the popliteal lymph node (PLN). Thus, we injected FITC-dextran into the footpad of TβRII^iΔLEC^ mice [19] 4 weeks after the administration of Tx (Fig. 3a). Five minutes later, FITC-dextran was found to be excreted in the PLN and reached the upper limbs of the control mice. On the other hand, less amount of FITC-dextran fluorescence could be observed in the lymphatic vessels upstream of the PLN around the thigh from TβRII^iΔLEC^ mice (Fig. 3b). FITC-dextran administrated into footpad might not be able to drain into the lymphatic vessels beyond the PLN due to impaired lymphatic function in TβRII^iΔLEC^ mice. We further explored lymphatic vessel dysfunction in TβRII^iΔLEC^ mice using a tumor lymphatic metastasis model. When LLC cells that constitutively express eGFP (LLC-GFP) were inoculated into the footpad of the mice 3 weeks after the administration of Tx (Fig. 3a), metastasis to PLN was noted 1 week after the transplantation of LLC cells (Fig. 3c). Metastasis of LLC cells was observed in 85.7% of control mice, whereas it was observed in only 27.3% of TβRII^iΔLEC^ mice treated with Tx, which significantly reduced compared to the control (*P* = 0.013; chi-square test) (Fig. 3d). Additionally, immunofluorescence staining was performed to confirm the presence of LLC-GFP cells in PLNs from wild-type mice (Fig. 3e). GFP-positive LLC cells were detected in the PLN stained with an anti-VEGFR3 antibody, demonstrating that lymphatic metastasis took place in the PLN. These results indicated that TGF-β signaling plays a key role in the maintenance of the lymphatic drainage function and that it promotes tumor lymphatic metastasis.

### TβRII^iΔLEC^ mice treated with Tx showed dilated tumor lymphatic vessels

**Fig. 4:**
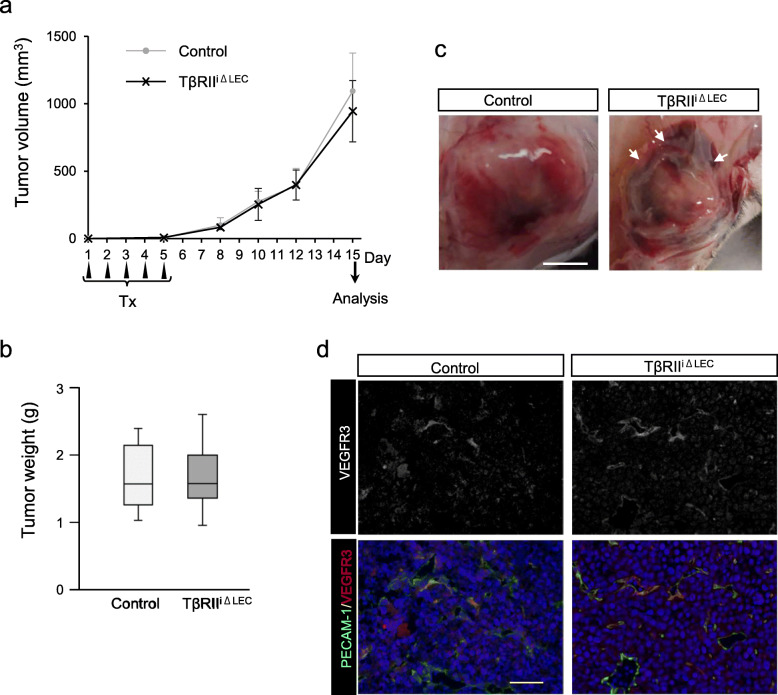
LEC-specific TβRII deletion does not affect tumor growth. **a** LLC cells were subcutaneously transplanted into Tx-treated control or TβRII^iΔLEC^ mice. Tumor size was measured from above the skin at the indicated days. Tumor volumes were calculated using the formula: length × width × width × 0.5. The data presented are the means ± S.D. (*n* = 6) **b** Tumor weight 15 days after LLC transplantation. (*n* = 6) **c** Appearance of xenografted tumors transplanted into control or TβRII^iΔLEC^ mice 15 days after LLC transplantation. TβRII^iΔLEC^ mice had tissue fluid accumulation around tumor (arrows). Representative images are shown. Scale bar: 5 mm. **d** Immunohistostaining of the tumor sections with anti-PECAM-1 (green) and anti-VEGFR3 (red) antibodies. Sections were counterstained with DAPI (nucleus, blue). Scale bar: 50 μm

To investigate whether deletion of the *TβRII* gene in LECs affects tumor growth, TβRII^iΔLEC^ mice were treated with tamoxifen consecutively for 5 days before a subcutaneous injection of 2.5 × 10^5^ LLC cells into their dorsal region. Figure 4a shows a comparison of the tumor volume between TβRII^iΔLEC^ and control mice 2 weeks after injection. There were no differences in tumor growth or tumor weight (Fig. 4b) between the two groups. However, accumulation of tissue fluid around tumors from TβRII^iΔLEC^ mice could be observed although the gross morphology of tumors between two groups was quite similar (Fig. 4c). Abundant VEGFR3-positive lymphatic vessels were observed in the tumors from TβRII^iΔLEC^ mice (Fig. 4d).

**Fig. 5:**
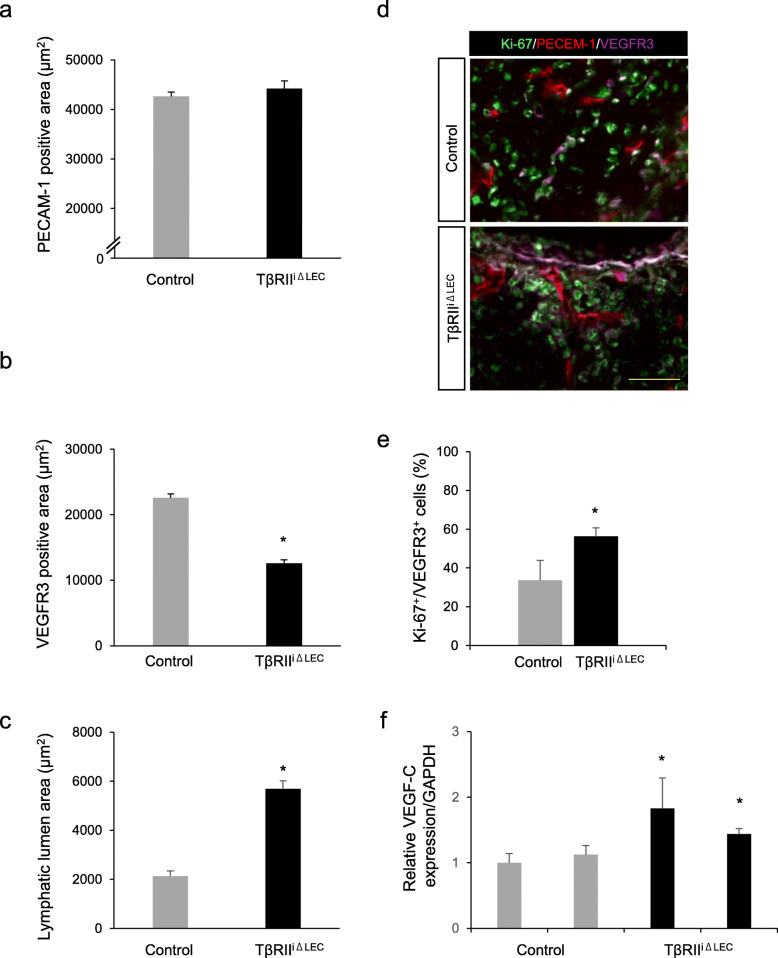
LEC-specific TβRII deletion potentiates lymphangiogenesis. **a–c** Image-based analysis of PECAM-1-positive, VEGFR3-positive, or lymphatic lumen areas. There was no significant difference in the average of PECAM-1-positive area between TβRII^iΔLEC^ (*n* = 3) and control mice (*n* = 3) (**a**). The average of the VEGFR3-positive area (**b**) and the lymph lumen area surrounded by VEGFR3 (**c**) in TβRII^iΔLEC^ mice (*n* = 3) was significantly higher than that in control mice (*n* = 3). **d** Immunohistostaining of the tumor-derived sections used in Fig. 4 with anti-Ki-67 (green), and anti-PECAM-1 (red), and anti-VEGFR3 (purple) antibodies. Scale bar: 50 μm. **e** Image-based analysis of Ki-67-positive LECs (percentage of Ki-67^+^ cells/VEGFR3^+^ cells). **f** qPCR analysis of tumor tissue from control and TβRII^iΔLEC^ mice (*n* = 2 per genotype). Expression levels of VEGF-C gene are depicted as fold induction relative to the expression levels of control mice. The data presented are the means ± S.D. (*n* = 3)

To determine the differences in blood and lymphatic vessel structures between control and TβRII^iΔLEC^ mice, we analyzed the sections that were stained with anti-PECAM-1 and anti-VEGFR3 antibodies for blood and lymphatic vessel structures, respectively. No differences in PECAM-1-positive area between control and TβRII^iΔLEC^ mice were observed in blood vessel structures (Fig. 5a), whereas the VEGFR3-positive areas were remarkably reduced in TβRII^iΔLEC^ mice (Fig. 5b). Additionally, lymphatic lumens in TβRII^iΔLEC^ mice were larger than those in the control mice (Fig. 5c), indicating upregulation of lymphangiogenesis. Inhibition of TGF-β signaling has been reported to promote lymphangiogenesis and LEC proliferation in the presence of VEGF-C. Therefore, we investigated endothelial cell growth and VEGF-C expression in the tumor tissue. Immunofluorescent staining of tumor tissues showed that Ki-67-positive lymphatic endothelial cells were increased in TβRII^iΔLEC^ mice (Fig. 5d, e), and the expression level of VEGF-C was significantly increased in the tumor tissue of TβRII^iΔLEC^ mice compared to the control mice (Fig. 5f). These data indicated that LECs in TβRII^iΔLEC^ mice might proliferate and form dilated lumens due to lack of TGF-β signaling under the control of VEGF-C.

## Discussion

Lymphangiogenesis is regulated by various cytokines, some of which control angiogenesis, such as vascular endothelial growth factors (VEGFs). TGF-β is not only implicated in angiogenesis and promotes blood vessel maturation [20], but also known to regulate lymphangiogenesis in a context-dependent manner [8, 9]. James et al. showed that LEC-specific deletion of the *TβRII* gene in embryos caused a marked decrease in lymphatic sprouting and remodeling during mouse development, when they injected Tx into pregnant dams at E12.5 and analyzed embryos at E14.5 [8]. Lymphatic progenitor cells start to bud from the cardinal vein at E10.5, and form lymph sacs at E11.5 [21]. Therefore, we administrated Tx to pregnant dams at E10.5 and E11.5 when the early stage of lymphatic differentiation and then analyzed at E13.5. Although there was not any alteration with respect to lymphatic cell differentiations from venous endothelial cell in embryos from TβRII^iΔLEC^ mice, we could see dilated lymphatic vessels in their embryos as reported by James et al. (Fig. 1b, c). As previously reported [20], TGF-β signaling might not be involved in the fate determination of LECs to establish lymphatic network formation. However, TGF-β signaling might play an important role in maintenance of the lymphatic vessel structure. In the mouse model for chronic peritonitis, the TGF-β kinase inhibitor increased LYVE-1-positive area [9]. They also showed lymphangiogenesis could be enhanced by VEGF-C secreted from inflammatory macrophages. The tumors from TβRII^iΔLEC^ mice also increased the area of VEGFR3-positive lymphatic lumen. This phenomenon might be due to the fact that LECs escaping from TGF-β signal proliferate in the presence of VEGF-C from the tumor microenvironment (Fig. 5e) [22]. Since the role of TGF-β signaling in LECs seems to be context-dependent, it would be necessary to analyze the crosstalk of TGF-β signal with other signaling pathways in future.

In this study, we used anti-LYVE-1 and anti-VEGFR3 antibodies to detect LECs (Figs. 1c and 2b, e), although there are several available antibodies that recognize lymphatic vessels. The anti-LYVE-1 antibody is useful to detect lymphatic vessel structures in fetal skin and tissue from adult mice. However, the anti-LYVE-1 antibody is not available in some cases because it can also recognize embryonic hematopoietic stem cells [23] and macrophages [24] in tumor microenvironment. Thus, we used the anti-VEGFR3 antibody in tumor tissues (Figs. 4d and 5d, Supplemental Figure 1). Podoplanin is another lymphatic specific maker [25], but it is also used as a cancer biomarker [26] (Supplementary Figure 1).

Cancer metastasis responsible for the death of patients with cancer is a hallmark of malignant tumors [27] [28]. Since cancer cells metastasize to distant organs through blood and lymphatic vessels, it is very important to understand how tumor angiogenesis and lymphangiogenesis are regulated in the human body. Tumor lymphatic vessels connect primary tumor cells and lymph nodes. Consequently, cancer cells invade the lymph nodes to move to other organs [29]. In the present study, we found that TGF-β signaling is involved in lymphatic drainage, in addition to confirming structural abnormalities of lymphatic vessels by the loss of LEC-specific TGF-β signaling. The quantitative analysis to evaluate the effect of TGF-β on lymphatic flow might be needed [30], but it is interesting that deletion of TGF-β signaling suppressed tumor lymphatic metastasis by reducing lymphatic drainage [31] (Fig. 3). TGF-β is abundant in the tumor microenvironment and enhances motility and metastasis of cancer cells. Our results suggest that the abundant TGF-β may act on lymphatic endothelial cells to promote tumor metastasis. Thus, the inhibition of TGF-β signaling by small compounds, antibodies, Fc-chimeric receptors [32], or RNA interference may block tumor metastasis via lymphatic vessels [33] [34].

## Conclusions

LEC-specific TβRII knockout mice showed that TGF-β signaling promotes lymphatic drainage. Furthermore, TGF-β enhances tumor metastasis via lymphatic vessels. Therefore, blockage of TGF-β signaling might inhibit tumor metastasis targeting the lymphatic vessels.

## Supplementary Information

**Additional file 1: Supplementary Figure 1.** Antibodies that recognize LECs in tumor tissue. LLC cells were subcutaneously transplanted into Tx-treated control TβRII^F/F^ mice, and 15 days after LLC injection, fresh frozen sections were prepared. Immunohistostaining with anti-podoplanin (PDPN, green), anti-LYVE-1 (red) and anti-VEGFR3 (blue) was performed. Scale bar: 100 μm

## Data Availability

All data generated and/or analyzed during the current stud are available from the corresponding author on reasonable request.

## Abbreviations

ALK: Activin-like kinase
cDNA: Complementary deoxyribonucleic acid
DMEM: Dulbecco’s modified Eagle’s medium
GFP: Green fluorescent protein
FCS: Fetal calf serum
FITC-dextran: Fluorescein isothiocyanate-dextran
LLC: Lewis lung carcinoma
LEC: Lymphatic endothelial cell
TGF-β: Transforming growth factor β
VEGF: Vascular endothelial growth factor
VEGFR: Vascular endothelial growth factor receptor
