# Supplementary material for: Endothelial-specific depletion of TGF-β signaling affects lymphatic function
Source: Inflamm Regen. 2021 Dec 1;41:35. doi: 10.1186/s41232-021-00185-4 (PMC8638105; doi:10.1186/s41232-021-00185-4)
Supplement: Supplementary file 1 — Additional file 1: Supplementary Figure 1. Antibodies that recognize LECs in tumor tissue. LLC cells were subcutaneously transplanted into Tx-treated control TβRIIF/F mice, and 15 days after LLC injection, fresh frozen sections were prepared. Immunohistostaining with anti-podoplanin (PDPN, green), anti-LYVE-1 (red) and anti-VEGFR3 (blue) was performed. Scale bar: 100 μm [file 41232_2021_185_MOESM1_ESM.docx]

**Supplementary Figure 1: Antibodies that recognize LECs in tumor tissue.** LLC cells were subcutaneously transplanted into Tx-treated control TβRII^F/F^ mice, and 15 days after LLC injection, fresh frozen sections were prepared. Immunohistostaining with anti-podoplanin (PDPN, green), anti-LYVE-1 (red) and anti-VEGFR3 (blue) was performed. Scale bar: 100 µm
